# Local seismic network for monitoring of a potential nuclear power plant area

**DOI:** 10.1007/s10950-015-9534-8

**Published:** 2015-11-05

**Authors:** Timo Tiira, Marja Uski, Jari Kortström, Outi Kaisko, Annakaisa Korja

**Affiliations:** Institute of Seismology, Department of Geosciences and Geography, University of Helsinki, Helsinki, POB 68, FIN-00014 Finland

**Keywords:** Seismology, Seismic monitoring, Seismicity, Tectonics, Seismic hazard, Nuclear power plant

## Abstract

This study presents a plan for seismic monitoring of a region around a potential nuclear power plant. Seismic monitoring is needed to evaluate seismic risk. The International Atomic Energy Agency has set guidelines on seismic hazard evaluation and monitoring of such areas. According to these guidelines, we have made a plan for a local network of seismic stations to collect data for seismic source characterization and seismotectonic interpretations, as well as to monitor seismic activity and natural hazards. The detection and location capability of the network were simulated using different station configurations by computing spatial azimuthal coverages and detection threshold magnitudes. Background noise conditions around Pyhäjoki were analyzed by comparing data from different stations. The annual number of microearthquakes that should be detected with a dense local network centered around Pyhäjoki was estimated. The network should be dense enough to fulfill the requirements of azimuthal coverage better than 180° and automatic event location capability down to ML ∼ 0 within a distance of 25 km from the site. A network of 10 stations should be enough to reach these goals. With this setup, the detection threshold magnitudes are estimated to be ML = −0.1 and ML = 0.1 within a radius of 25 and 50 km from Pyhäjoki, respectively. The annual number of earthquakes detected by the network is estimated to be 2 (ML ≥ ∼ −0.1) within 25 km radius and 5 (ML ≥ ∼−0.1 to ∼0.1) within 50 km radius. The location accuracy within 25 km radius is estimated to be 1–2 and 4 km for horizontal coordinates and depth, respectively. Thus, the network is dense enough to map out capable faults with horizontal accuracy of 1–2 km within 25 km radius of the site. The estimation is based on the location accuracies of five existing networks in northern Europe. Local factors, such as seismic noise sources, geology and infrastructure might limit the station configuration and detection and location capability of the network.

## Introduction

**Fig. 1:**
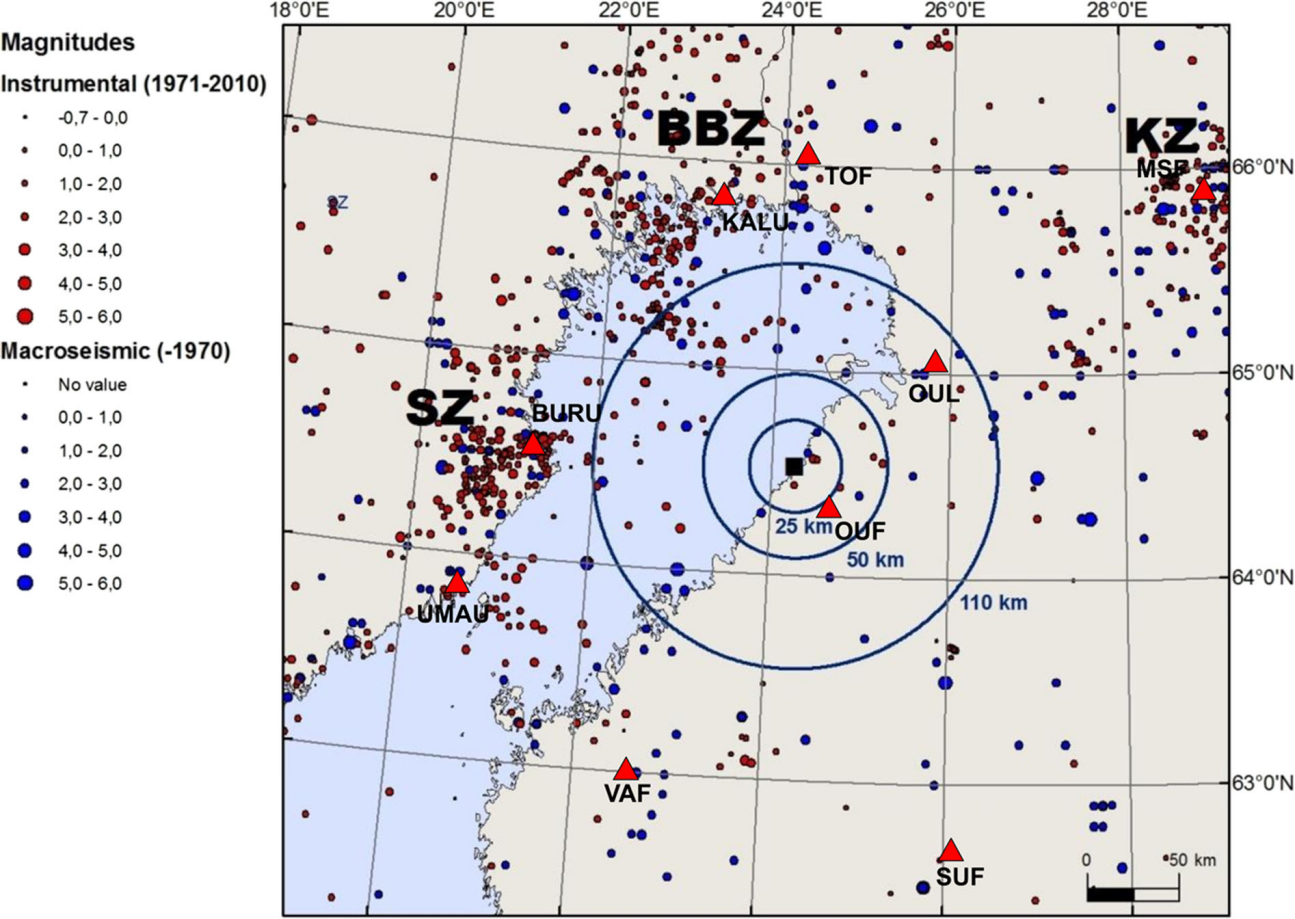
A seismicity map of Pyhäjoki area for the period 1626–2010. Macroseismic and instrumental epicenters are denoted by *blue and red dots*, respectively. Macroseismic data is from Mäntyniemi and Ahjos (1990). Three concentric circles have a 110, 50, and 25 km radii from Pyhäjoki (*black square*). Seismicity zones: *SZ* Skellefteå; *BBZ* Bothnian Bay-Finnmark; *KZ* Kuusamo

Sites of nuclear power plants must be evaluated for seismic risk and monitored for seismicity (IAEA, 3.30, 2010). A new nuclear power plant is planned to be constructed at Hanhikivi, Pyhäjoki, Northern Ostrobothnia. Pyhäjoki is situated in the Central part of the Fennoscandian Shield, a region characterized by low intraplate seismicity (Fig. 1). European Union’s directive (2009/71/EURATOM 9) recommends the operators to follow IAEA’s guidelines on seismic hazard evaluation and monitoring of the area. According to IAEA (3.30, 2010) guidelines, a network of sensitive seismographs having a recording capability for microearthquakes should be installed to acquire more detailed information on potential seismic sources when a nuclear power plant site is evaluated. The operation period of the seismograph network should be long enough to obtain data for seismotectonic interpretation (IAEA, 3.30, 2010), and the monitoring of natural hazards shall commence no later than the start of construction and shall continue up until decommissioning (IAEA, 5.1, 2003). Strong motion accelerographs should be installed permanently within the site area. The data processing, reporting, and network operation are advised to be linked to the regional and/or national networks. The IAEA (2010) sets special requirements for intraplate regions, where longer observation periods should be used, capable faults and sources at larger distances should be taken into consideration, and maximum magnitudes should be assumed larger.

When a dense, local seismic network is set up, numerous small earthquakes are expected to be recorded within a relatively short time period. Local seismic networks have better location accuracy and source depths can be evaluated more reliably than with more sparse regional networks. Seismotectonic interpretation and seismic hazard evaluation can be improved with a larger amount of accurately located earthquakes.

In the preliminary geological and geophysical studies of the Pyhäjoki area (Kuivamäki et al. 2011; Korja et al. 2010; Pihlaja et al. 2011; Poutanen et al. 2011 and Putkinen and Valpola 2011) no capable faults could be identified within a radius of 25 km. The data sets did not, however, cover all of the offshore areas and the accuracy of epicenter locations close to Pyhäjoki was rather low (Kukkonen 2011). In order to attach seismic events to single faults or shear zones, more events with better hypocenter location accuracy are sought for.

The focus of this study is to outline a plan for a local seismograph network centered around Pyhäjoki. The network should serve to collect data for seismic source characterization and seismotectonic interpretations as well as to monitor seismic activity and natural hazards throughout the lifetime of the planned facility. First, an ideal network configuration without the restrictions of infrastructure, geographical barriers or geological boundaries is searched for. Modifications to the ideal configuration are expected in the deployment phase.

There are many methods for estimation of the lowest magnitude of events that a seismic network is able to detect (Sereno and Bratt 1989; Gomberg 1991; Woessner and Wiemer 2005; Schorlemmer and Woessner 2008). They are usually used to evaluate the performance of an existing seismic network or completeness of an earthquake catalog. We try to estimate what kind of network is needed to fulfill certain level of performance. We use data from existing networks of different sizes in the same larger region to calculate estimated spatial distribution of the magnitude of the smallest detectable earthquake. An estimate for accuracy of the hypocentral locations produced by the planned network is formed by studying performance of the existing networks of about similar size in the region, and by comparing the geographical layout of the planned network to those of the existing networks. The event detection and location performance of a local seismic network around Pyhäjoki will be simulated and recommendations on the optimal configuration of the network will be given. The simulations are based on automatic event bulletin data sets published by the Institute of Seismology, University of Helsinki (ISUH). The activity rate of microearthquakes below the current detection threshold is estimated by using the Fennoscandian earthquake catalog (FENCAT; Ahjos and Uski 1992). The azimuthal coverage and threshold magnitude is computed for different types of station configurations and the results are presented as maps.

## Noise conditions and their effect on earthquake detection and location capability

The location capability of a seismic network depends on the detection capability, on the background noise level and on the geometrical configuration of the network. The event detection capability of a seismic station depends strongly on its background noise level and signal to noise ratio (SNR) (Bolt 1976). In automatic detection programs, the amplitude generated by an event is required to be larger than the background noise by a preset threshold (Ambuter and Solomon 1974). Lower threshold values can be used if the noise conditions are favorable or the risk of increased number of false detections is manageable (Bratt et al. 1990).

**Fig. 2:**
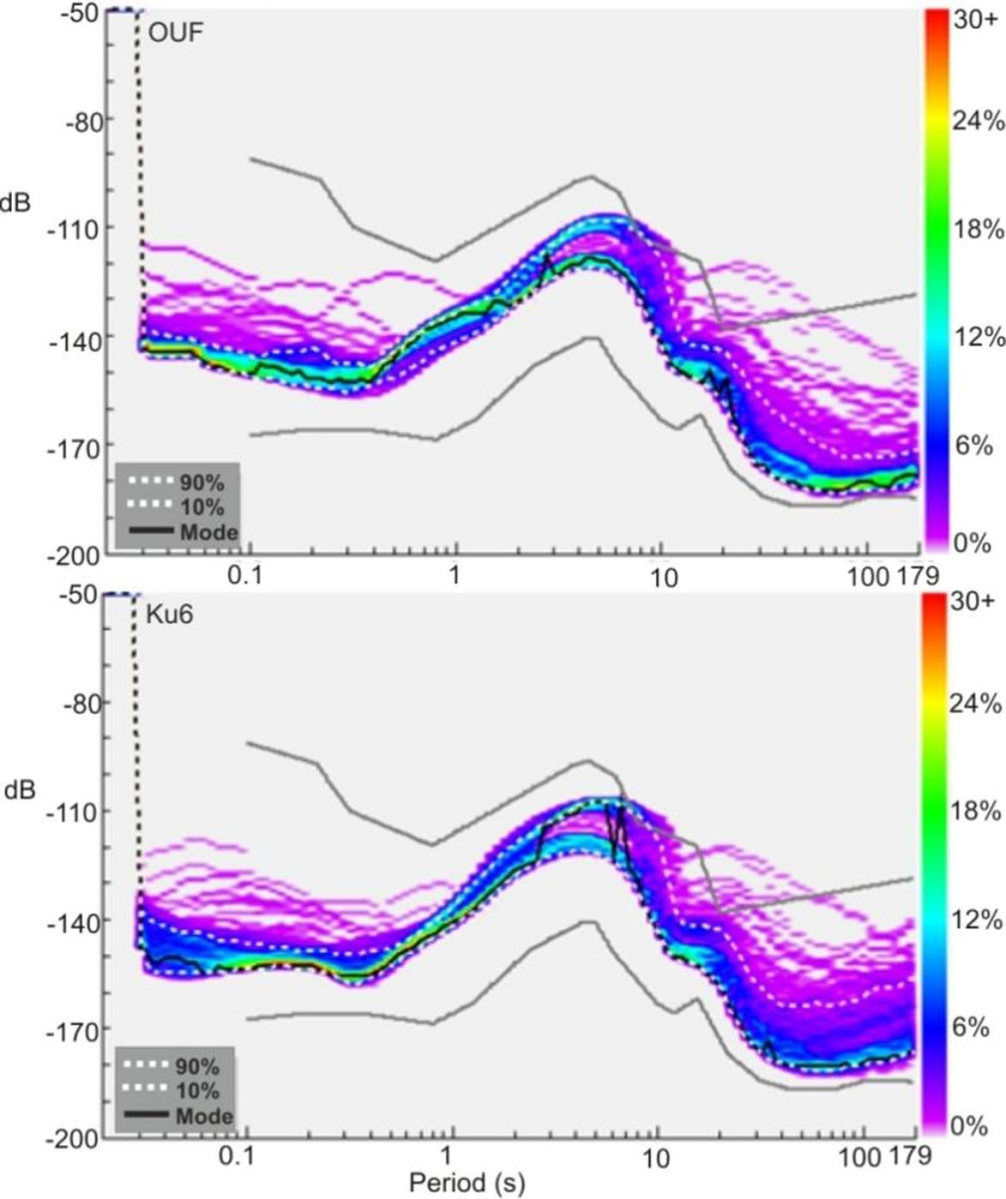
Background noise level at stations OUF and KU6 displayed as power spectral density functions. The power spectral densities are calculated from 1 week of continuous recording for the same time period at both stations. The *gray lines* denote the global average of low and high noise level models (Peterson 1993). The *white dashed lines* denote 10th and 90th percentiles. The *black dashed line* shows the median

Background noise conditions around Pyhäjoki are analyzed by using existing waveform data sets from the closest permanent station OUF, situated 29 km east of Pyhäjoki. The calculations are performed with the PQLX software (McNamara and Boaz 2005), which uses the power spectral density technique of McNamara and Buland (2004). The results are compared with those from the station KU6 (Fig. 2). Note that the average of low period (high frequency) noise is slightly higher at OUF but its variation is smaller than at KU6. KU6 is part of the FNSN permanent station network. It belongs also to the Kuusamo local network (KULN) (Uski et al. 2011) in north-eastern Finland. Most of the low magnitude (M_L_ < 1) earthquakes used in the current study have been detected by KULN temporary network. The typical station spacing of KULN network is 40 km. Although the noise conditions are roughly similar at the two stations, OUF has a bit higher average noise level than KU6 at high frequencies (period below 0.1 s), which are used in detecting weak signals at short distances. The local magnitude scale (Uski and Tuppurainen 1996) is calibrated to Richter’s reference curve at 60 km.

**Fig. 3:**
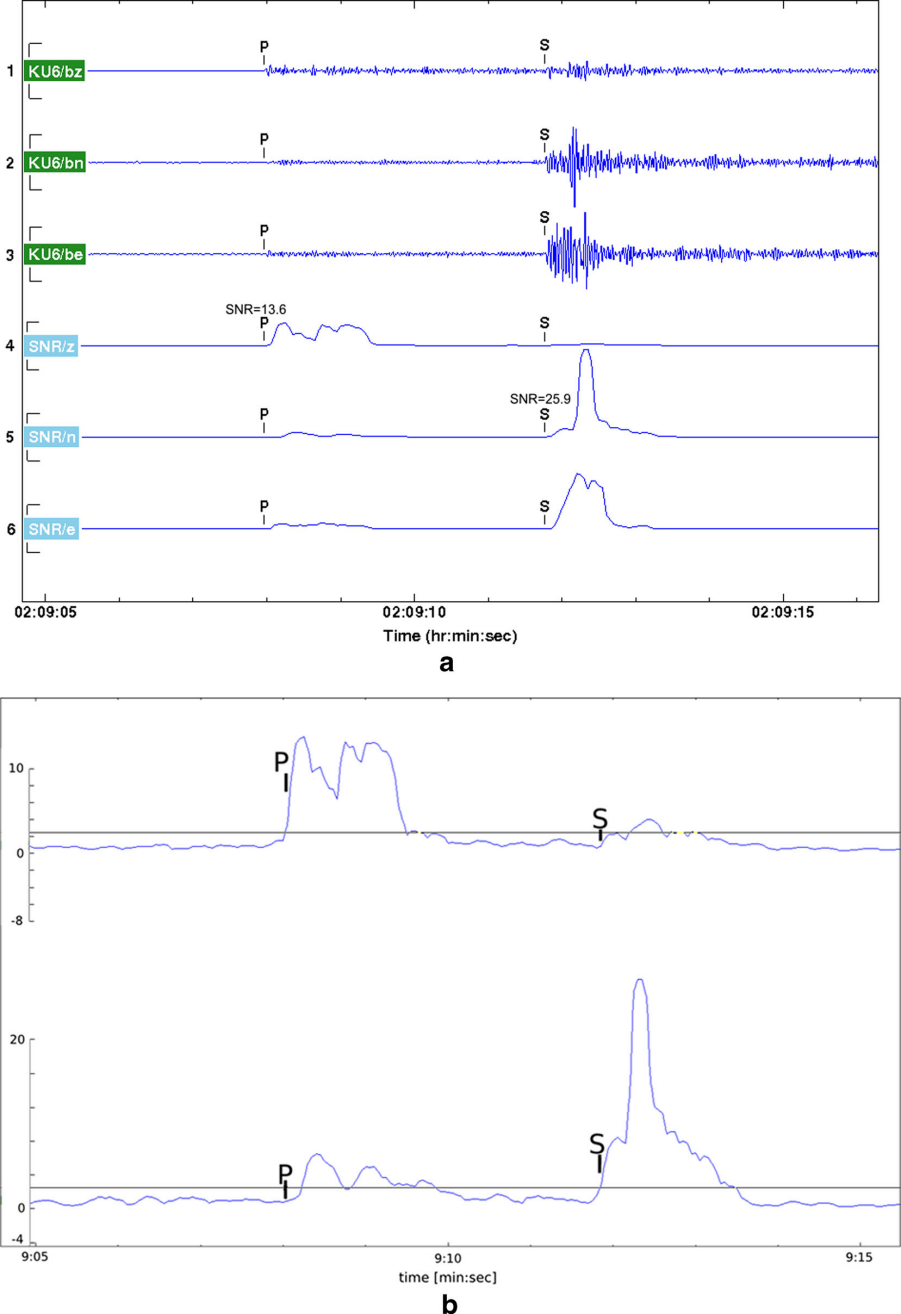
**a** An example showing 3-C recording of a low magnitude earthquake (ML −0.1) at KU6, 21 km from the source. The topmost three traces (vertical, north, and east) are filtered (band-pass 10–30 Hz) recordings and the bottom three traces are corresponding SNR traces of the waveforms. **b** Partial enlargement of vertical Z and horizontal N traces in **b**. The *black lines* show the detection threshold

**Fig. 4:**
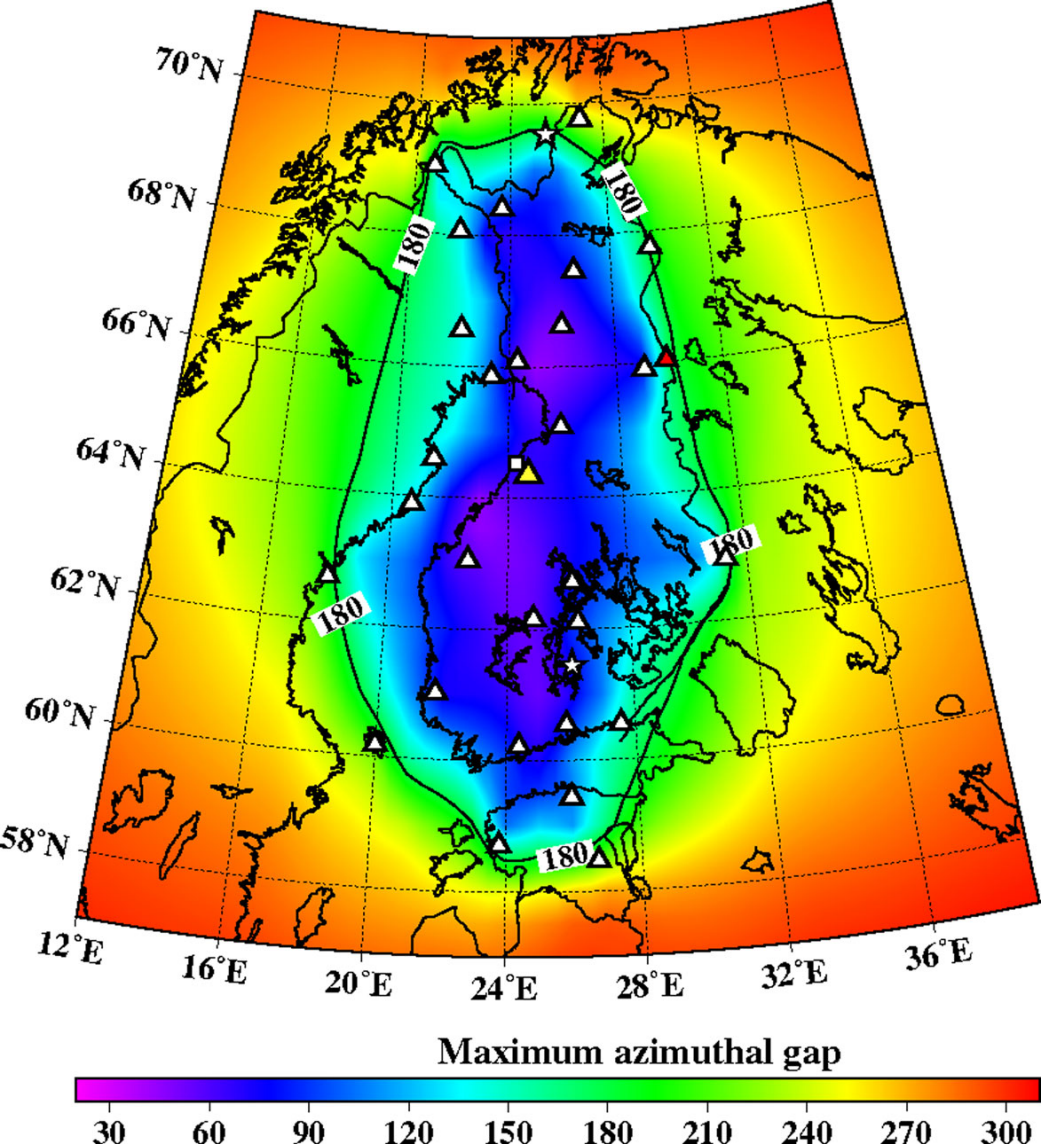
On-line seismic stations used by ISUH in automatic event processing on a map of maximum azimuthal gap. A *triangle* denotes 3-C station and a star seismic array. Stations OUF and KU6 are marked with *yellow* and *red* fill, respectively. Pyhäjoki plant area is marked with a *square*

Figure 3a shows an example of filtered three-channel (3-C) recordings and their SNR at KU6. Note that SNR is well above the detection threshold (2.2) used in automatic analysis (Fig. 3b). The studied event is a local earthquake of M_L_ −0.1 at a distance of 21 km from KU6. It is the weakest event of which both the P- and S-wave onsets have been automatically detected by KULN. Prior to detection, the signal was filtered with a band-pass filter of 10–30 Hz. Note that the SNR levels are well above the detection thresholds used in the automatic analysis (Fig. 4). Based on the roughly similar levels of background noise in the KULN and Pyhäjoki areas, the network around Pyhäjoki is expected to have similar ability to record small earthquakes and other seismic events as KULN.

## Automatic location system of ISUH

ISUH has an in-house-designed automatic data processing system, which utilizes the available on-line 3-C and array stations in Finland and in the neighboring countries (Fig. 4). The automatic detection routine suggests initial locations of seismic events with single station back azimuth determination and associates the initial locations with detections from other stations. At single 3-C stations the detection is based on software by Ruud and Husebye (1992), which uses the back azimuth determination and phase identification method of Roberts et al. (1989) to produce automated single-station event bulletins. The seismic array stations FINES and ARCES in southern Finland and in northern Norway, respectively, use processing methods and software developed by NORSAR (Bache et al. 1990) to produce single station event bulletins.

The single station results are combined with a program which continuously reads the locations from other single station bulletins. It calculates theoretical P- and S-wave arrival times for each station and for each source. Suitable P- and S-onset times are searched from initial detection logs using a chosen time window. If three or more stations have phases associated with the same source, they are run through location program HYPOSAT (Schweitzer 2001) to improve the source parameters. The back azimuth value, which is obtained from 3-C detectors and array detectors, is also used for location. This method allows the usage of low detection thresholds at single stations—a necessity in detecting weak signals. The relatively high rate of false alarms in single station detection logs is manageable with association rules.

The automatic event processing using this method started in 2007. Last significant upgrade to the system was in 2010 when the Swedish National Seismic Network (SNSN; Bödvarsson et al. 2006), operated by the University of Uppsala, provided six stations for the ISUH automatic on-line analysis. This improved the detection capability in the Finnish-Swedish border zone in Lapland and in the Bothnian Bay.

The station network that is currently used in automatic processing is shown in Fig. 4. At present, it consists of 17 stations operated by ISUH, 4 stations operated by the Sodankylä Geophysical Observatory, University of Oulu, 7 stations operated by University of Uppsala, 3 stations operated by the Geological Survey of Estonia, and 1 station operated by NORSAR, Norway.

Similar automatic location system with parameters suitable for local area network has been used to locate seismic events at KULN. Data from the local network in Pyhäjoki area will be processed with a similar system that is tuned for local conditions.

## Seismicity statistics for Pyhäjoki area

Pyhäjoki is a seismically quiet area located between three zones of increased seismic activity: Skellefteå (SZ) in the west, Bothnian Bay Zone–Finnmark (BBZ) in the north-west and Kuusamo (KZ) in the north-east (Fig. 1) (Korja et al. 2015). The earthquake observations from the area date back to the eighteenth century. The strongest historical earthquake took place in 1737 on the east coast of Bothnian Bay, 85 km south-west of Pyhäjoki. It has been assigned with a macroseismic magnitude of 4.1. No event of magnitude greater than 4.0 has been recorded in the area during semi-instrumental (1956–1970) and instrumental (1971–2011) era. Furthermore, only three instrumental events, with magnitudes (M_L_) ranging from 1.7 to 2.3, have been detected within 25 km of Pyhäjoki (Fig. 1). The earthquake information has been retrieved from FENCAT catalog (Institute of Seismology, University of Helsinki 2015; Ahjos and Uski 1992).

In the following, the annual number of microearthquakes that should be detected with a dense local network centered around Pyhäjoki is estimated. In order to calculate a Gutenberg-Richter (G-R) frequency-magnitude curve (Gutenberg and Richter 1944) representative for the area, the source region must be defined. The region must be large enough to include a sufficient number of earthquakes for statistical analysis, but small enough to exclude the neighboring regions with different seismotectonic characteristics. An area with a radius of 110 km around Pyhäjoki is estimated to fulfill the requirements (Fig. 1). From this, we have excluded seismicity along the southernmost part of the BBZ and the Skellefteå region in eastern coast of Sweden, the seismically most active area in Sweden, since those events belong to different seismotectonic environments. The study focuses on instrumentally recorded earthquakes because it is not straightforward to determine threshold magnitude (Mt) for historical data sets or to compare macroseismic and instrumental magnitudes. The earthquake information has been retrieved from FENCAT catalog (Institute of Seismology, University of Helsinki 2015; Ahjos and Uski 1992).

Frequency-magnitude distribution of earthquakes is generally approximated by the G-R-relation (Gutenberg and Richter 1944):

1 $$ lo{g}_{10}(N)=a-bM, $$

where *N* is the cumulative number of earthquakes with magnitude equal to or greater than *M* occurring in a specified space and time window. Intercept *a* measures the activity rate and slope *b* defines the ratio of small to large earthquakes.

Over long time periods and large spatial scales, the *b* values of tectonic earthquakes approximate 1.0, i.e., the distribution of earthquake size is invariant with respect to scale. The shorter the time interval or the smaller the area, the more the fit is degraded by insufficient data sampling. In continental interiors, the recurrence periods of large earthquakes may exceed the time interval studied. Furthermore, at low magnitudes the data may be incomplete due to the detection threshold of the seismic network.

In seismic hazard assessment, the G-R -relation has been used to predict the recurrence periods of rare large earthquakes from the number of weaker but more common events. Conversely, some studies have demonstrated that earthquake self-similarity extends at least down to magnitude ML ∼ 0 (Abercombie 1996 and references therein). These results suggest that the G-R -relation can also be applied in estimating the number of microearthquakes from the occurrence rate of stronger events (e.g., Häge and Joswig 2009). This approach is also used in this study to estimate the number of microearthquakes that could be detected with a dense local network around Pyhäjoki.

**Fig. 5:**
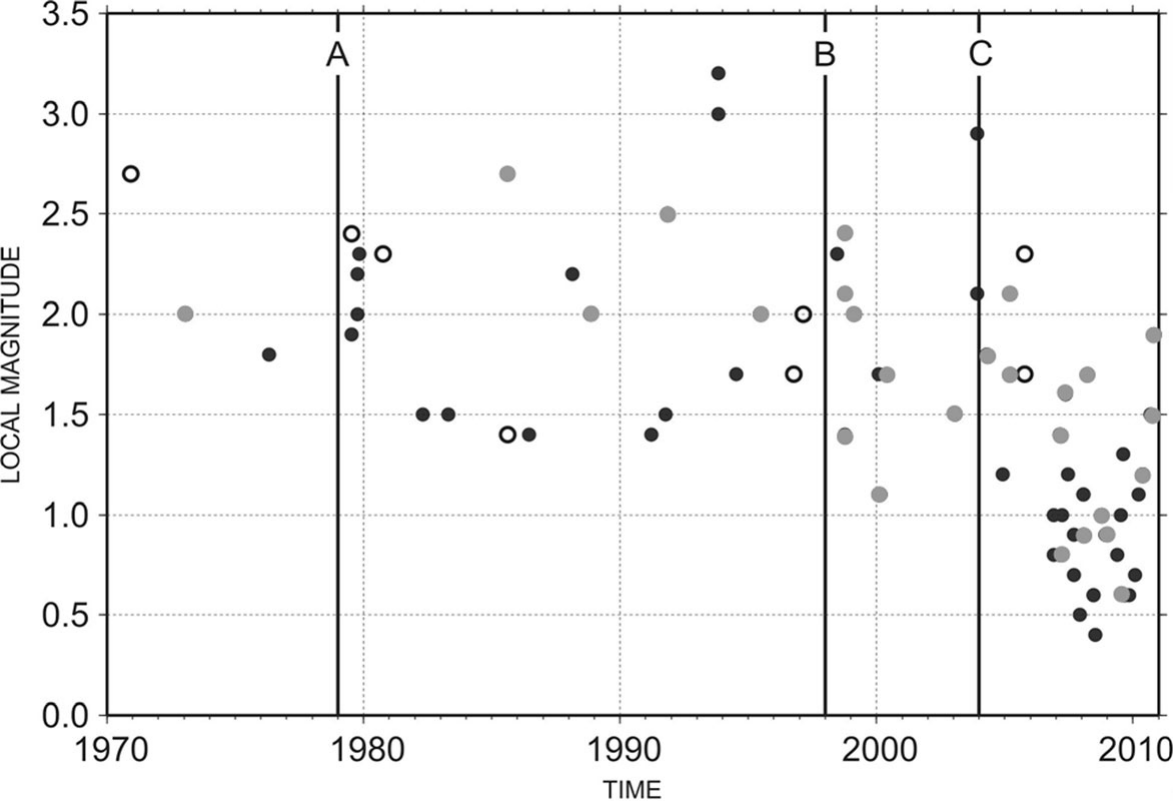
A time-magnitude distribution of earthquakes recorded within 110 km (*filled circles*) and 50 km (*open circles*) distance of Pyhäjoki during 1970–2010. *Gray filled circles* denote earthquakes which were excluded from the analysis since belong to different seismotectonic environments. The *letter symbols* mark the most significant changes in catalog completeness, see text for more details

Figure 5 shows a time-magnitude distribution of instrumental earthquakes located within a 110-km distance from Pyhäjoki. The plot reveals different levels of completeness in the local catalog. The first change (A) occurred in late 1970s when digital three-partite arrays in southern and central Finland became fully operational, allowing for systematic use of instrumental detection, location and magnitude determination methods. Modernization of SNSN, a dense digital network now covering all of Sweden, began in 1998 (B) and by the end of the same year, the upgrade of FNSN to a digital high-frequency network was completed. The most significant increase in number of events as well as decrease in observed Mt occurred around 2004 (C) when SNSN started providing parameter data to FENCAT. However, the majority of those microearthquakes are located in the Swedish side of the Bothnian Bay, at a distance of 50–110 km from Pyhäjoki (cf. Fig. 1). The time period of 1979–2011 was chosen for subsequent analysis because of an average constant seismicity above Mt of 1.6 and homogeneity of local magnitudes. To exclude earthquakes of a different seismotectonic zone (southernmost end of BBZ), events in area bordered by latitude 65.0 and longitude 23.5 in south-west edge were left out of the analysis.

**Fig. 6:**
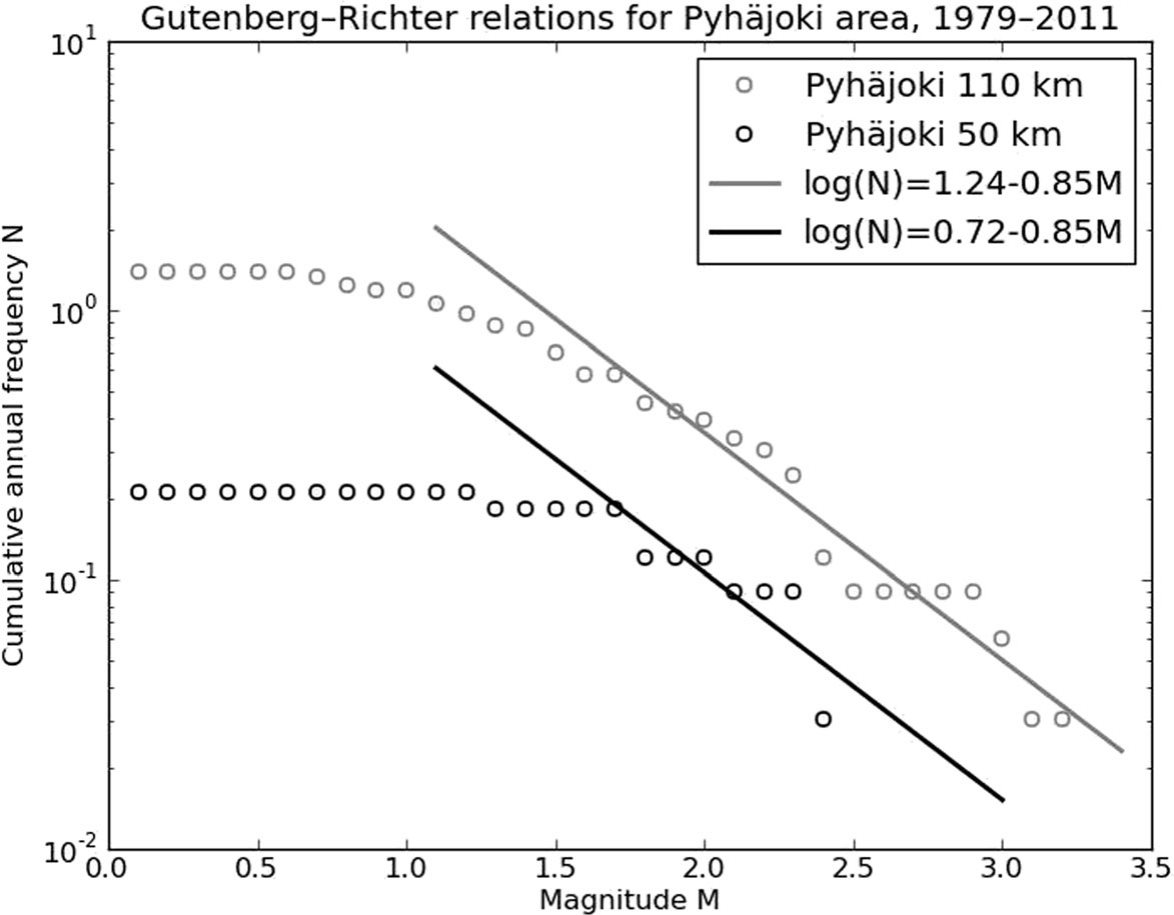
Magnitude-frequency curves for earthquakes recorded within 110 and 50 km radius of Pyhäjoki during 1979–2010. N is the cumulative annual number of earthquakes with magnitude M greater than or equal to a certain value. *Gray and black lines* show the *b* value determined for the 110 km data that is also applied to the 50 km data

Figure 6 shows the frequency-magnitude distribution of the earthquakes located within 110 and 50 km of Pyhäjoki during 1979–2010. The values a(110 km) = 1.24 ± 0.14 and b(110 km) = 0.85 ± 0.06 were obtained by linear least squares regression analysis with Mt = 1.7. The spatial coverage of seismicity is rather heterogeneous, as only 7 of the 70 events fall within the 50 km radius (cf. Fig. 1). However, by assuming that the obtained *b* value is a representative value of the whole volume, the activity rate of a(50 km) = 0.72 ± 0.12 is obtained. The catalog is complete only for magnitudes larger than 1.7, resulting in a dataset with a very narrow magnitude range, between *M* = 1.7 and *M* = 3.2. The accuracy of the fit is limited due to the scarcity of events of higher magnitude (ML ≥ 2.4). The expected rates of earthquake occurrences within 110, 50 and 25 km area of Pyhäjoki are given in Table 1.

**Table 1:** Annual frequency of earthquakes with magnitude M_L_ ≥ −1.0 within 110, 50, and 25 km radius of Pyhäjoki

M_L_	110 km	50 km	25 km
−1.0	123	37	9
−0.8	83	25	6
−0.6	56	17	4
−0.4	38	11	3
−0.2	26	8	2
0.0	17	5	1
0.2	12	4	1
0.4	8	2	1/2 years
0.6	5	2	1/2 years
0.8	4	1	1/4 years
1.0	2	1	1/5 years
2.0	1/3 years	1/10 years	1/38 years
3.0	1/20 years	1/68 years	1/270 years

## Simulation of automatic networks

**Fig. 7:**
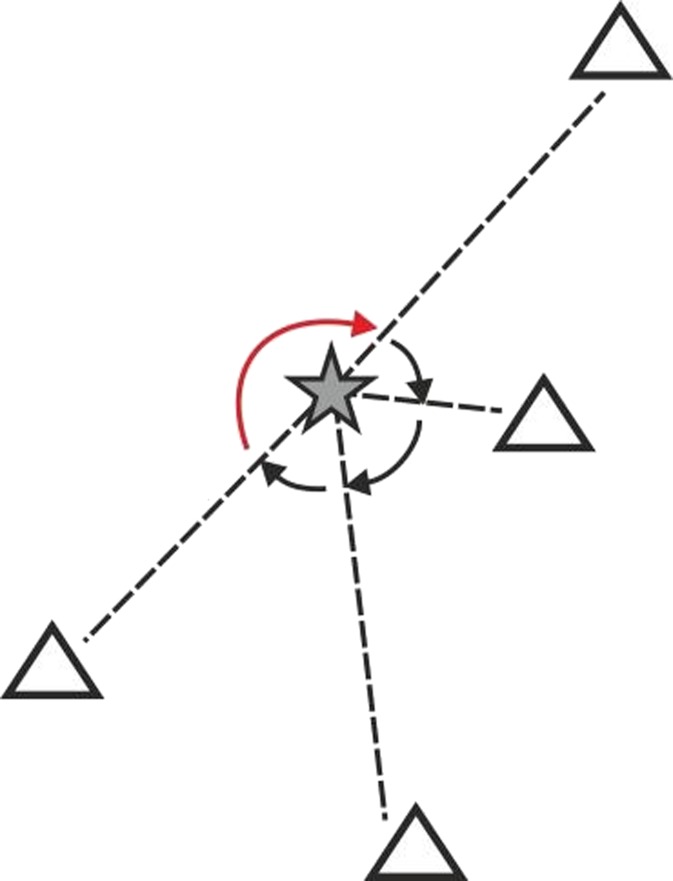
Azimuthal gap. An earthquake epicenter is denoted by star, seismic stations by triangles and epicenter-to-station paths by *dashed lines*. The *arrows* denote azimuthal gaps, the *red* one being the maximum gap

A modern local seismic network is designed to exploit automatic event detection and location routines. The location accuracy of the network depends mainly on its spatial configuration and SNR. The amplitude of seismic waves attenuates with distance. Thus the magnitude of the smallest detectable earthquake depends on the range of epicentral distances. The location accuracy is influenced by azimuthal gap. Azimuthal gap is the maximum angle separating two adjacent seismic stations, both measured from the epicenter of an earthquake (Fig. 7). If azimuthal gap is more than 180°, the location accuracy degrades significantly. The detection capability of a network can be evaluated based on the relationship between magnitude and observation distance.

**Fig. 8:**
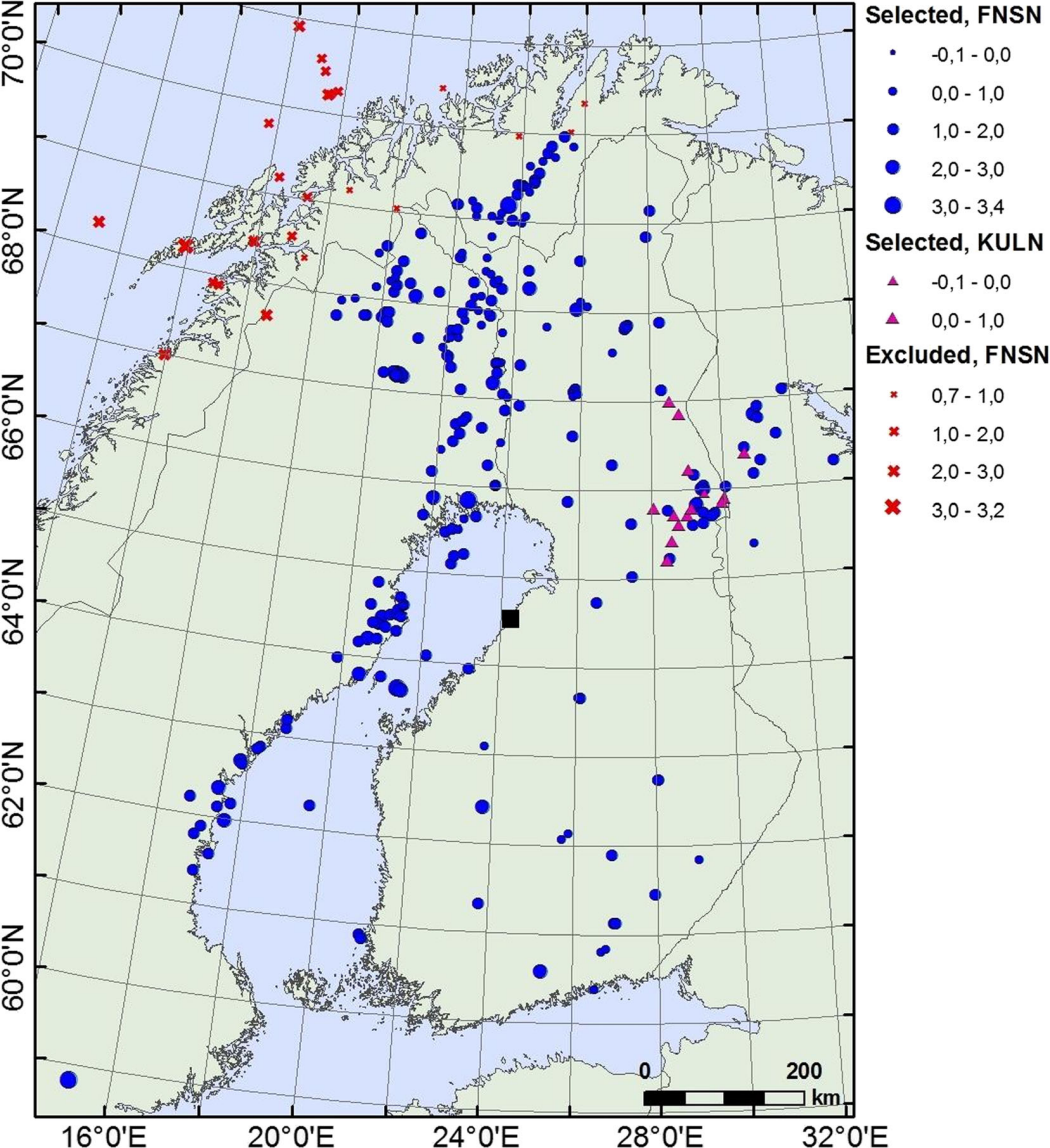
The data set of automatically located earthquakes from similar geological environments to Pyhäjoki. The events included in the study are marked with *dots* (FNSN) and *triangles* (KULN) and the events excluded with crosses. The magnitudes of the events are expressed with the proportional sizes of the symbols

**Fig. 9:**
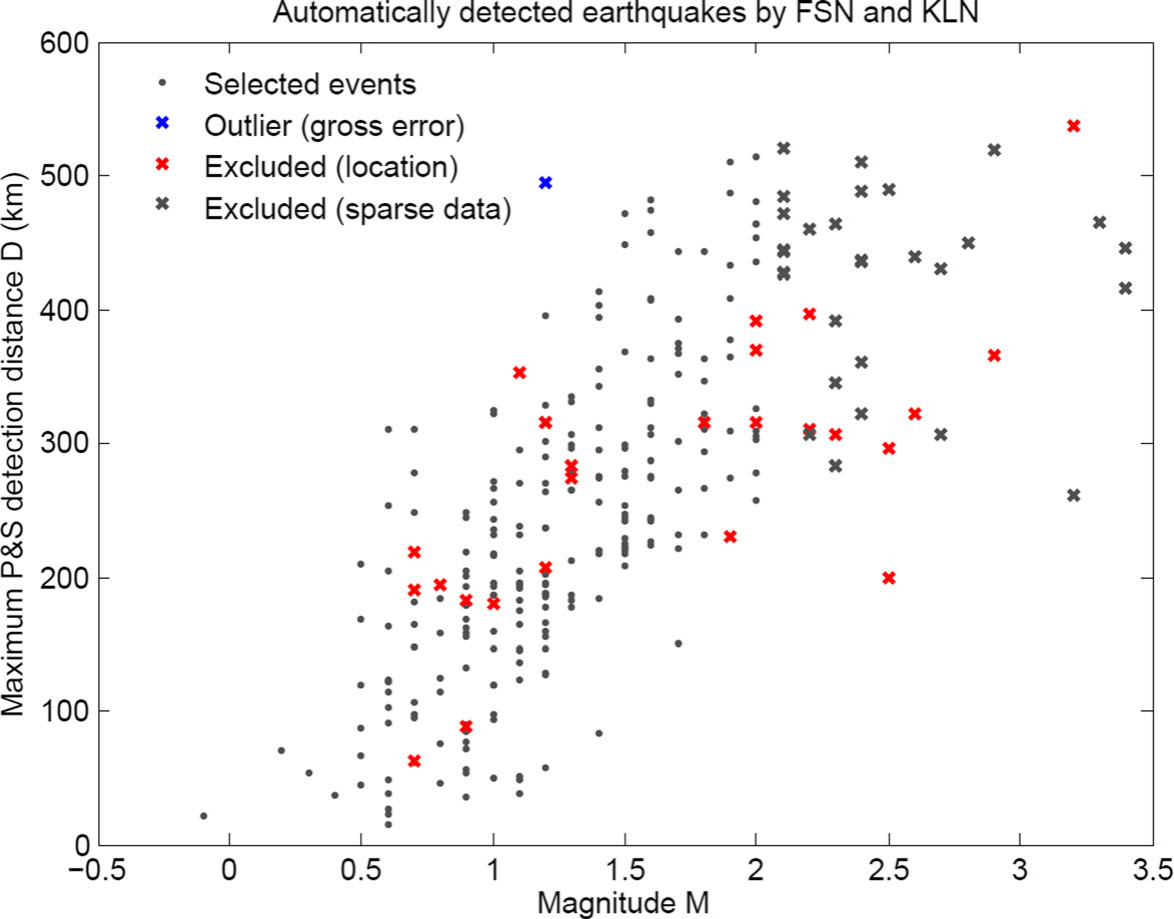
A magnitude-distance plot of the events in Fig. 8. The data included is presented with *gray dots* and the data excluded with crosses

In the following, the automatic location capability of Pyhäjoki network is investigated by analyzing the relation between magnitude and distance at which both P- and S-phases can be automatically detected. The underlying assumption is that Pyhäjoki area has similar attenuation characteristics and noise conditions to other parts of Finland. Preliminary studies of seismic noise conditions in the area support this assumption (Valtonen et al. 2012; Korja et al. 2010). The data comprise the earthquakes detected by FNSN supplemented with smaller magnitude earthquakes detected by KULN. The data set includes 259 earthquakes located within the Fennoscandian Shield (Figs. 8 and 9). First, for each automatically detected earthquake the largest epicentral distance where both P- and S-phases have been detected is selected. The maximum distance is associated with a manually reviewed magnitude. Then, gross error analysis is performed to weed out outliers from the data set (Wang et al. 2011). Only one event is excluded after the analysis (Fig. 9). There are only few events with maximum detection distance above 500 km in the data set because the automatic processing of FNSN data does not search for events with larger epicentral distance than 500 km. Most events larger than magnitude 2.0 in the region occur farther than 500 km from the stations. Consequently, the number of earthquakes at magnitudes larger than 2.0 in the database is small and their distance range is biased. Therefore, the analysis is limited to the events below magnitude 2.0 (Fig. 9).

Ground motion can be expressed in a simplified form with formula (see e.g. Joyner and Boore 1981; Liu and Tsai 2005):

2 $$ log(Y)=A+BM-lo{g}_{10}(R)+CR $$

where *Y* is ground motion, *M* is magnitude, *R* is distance to hypocenter and *A*, *B*, and *C* are coefficients. If ground motion is assumed to be constant for the detection threshold, the magnitude can be expressed with formula:

3 $$ M=blo{g}_{10}(D)+ aD+c $$

where *D* is distance from earthquake source, *M* is magnitude, *a* and *b* are coefficients of anelastic attenuation and geometrical spreading, and *c* is a baseline correction. The difference between epicentral and hypocentral distances is neglected since depth range of the earthquakes is small compared to epicentral distance.

**Fig. 10:**
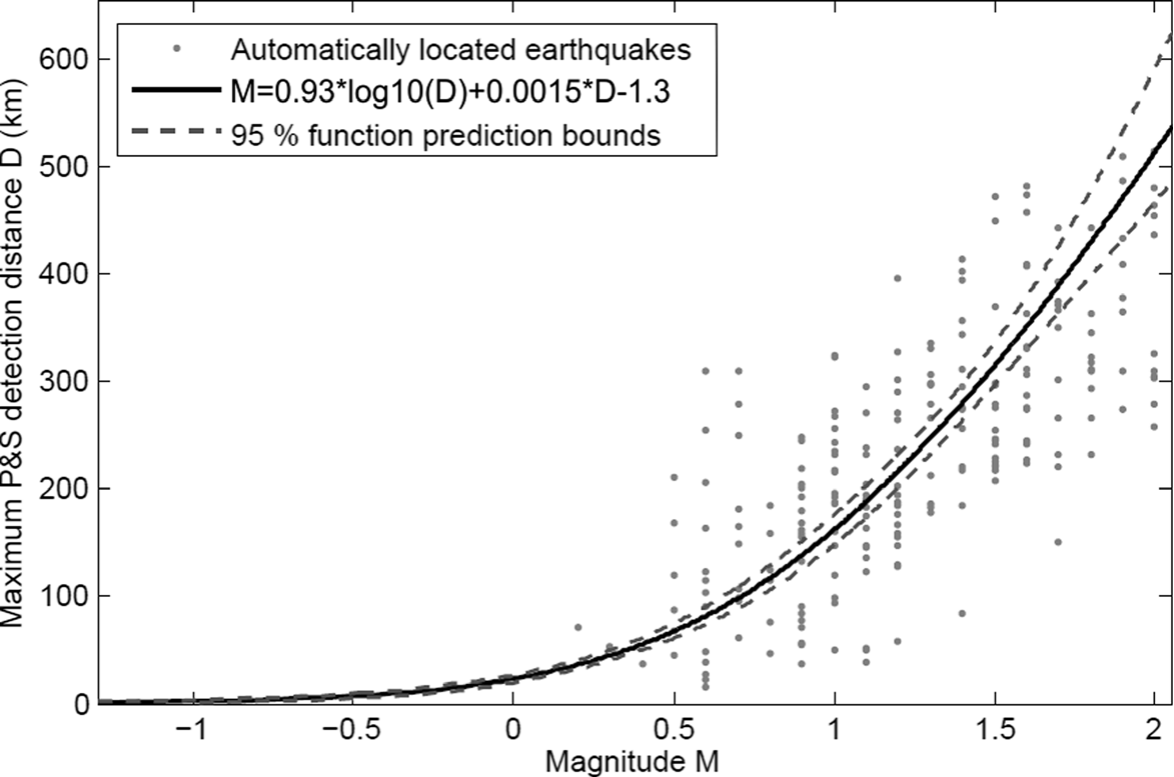
The calculated fit to the relationship between magnitude and maximum P- and S-wave detection distance. The fit is drawn with *black line* and the non-simultaneous function prediction bounds for 95 % values with *dashed lines*. The magnitude-distance -pairs are drawn with *gray dots*

The data are weighed according to the number of points in the magnitude bins. Bin width is 0.1 magnitude units. By fitting the function (2) with the least squares method to the data (Fig. 10), the following equation is obtained:

4 $$ M=0.93lo{g}_{10}(D)+0.0015D-1.3 $$

The capability to automatically detect seismic events can be tested with seismic network simulations. The relation (4) between magnitude and the maximum detection distance (Fig. 10) is applied to calculate the minimum detectable magnitude maps (Figs. 11, 12a, 13a, 14a, 15a, 16a, and 17a) for the study area.

**Fig. 11:**
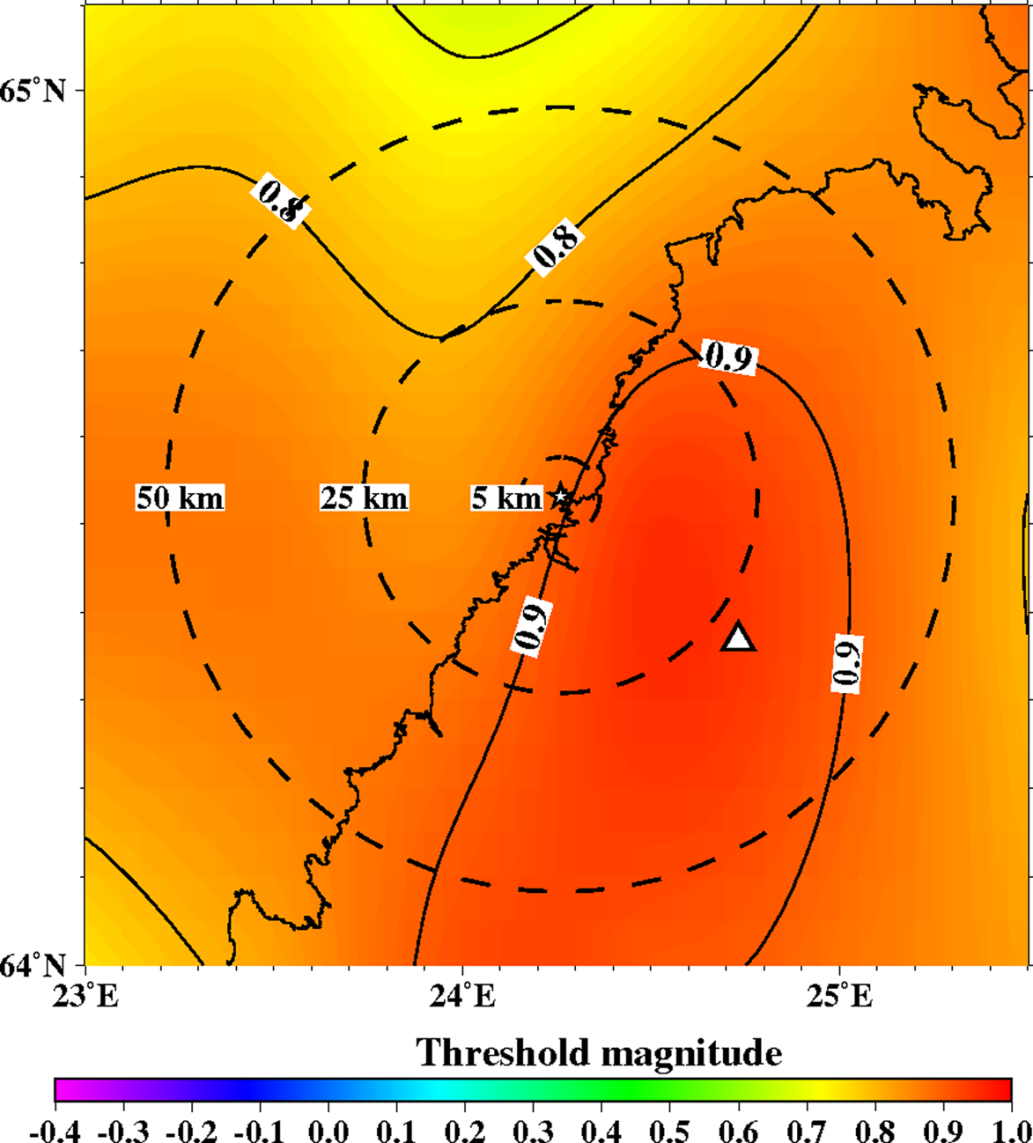
The minimum detectable magnitude by automatic location system for the study region using the current station configuration of FNSN. Isolines show minimum detectable magnitude. The *dashed circles* denote areas with radii of 5, 25 and 50 km from the power plant site, which is marked with a *star*. Permanent seismic station OUF is marked with a *triangle*

**Fig. 12:**
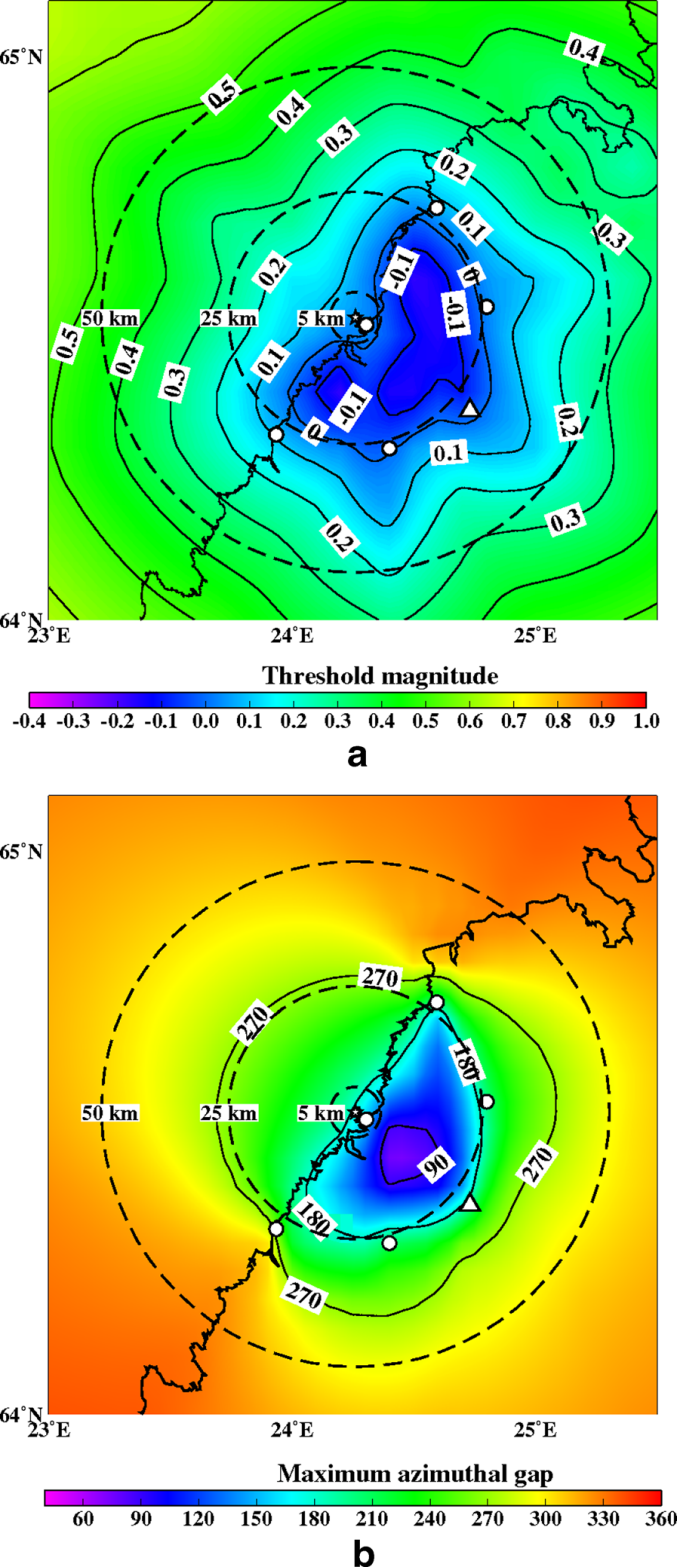
**a** The minimum detectable magnitude for the study region with a six station network (OUF+5 new ones). The new station sites are marked with *small circles*. Other abbreviations and explanations as in Fig. 11. **b** The maximum azimuthal gap for the study region with a six station network (OUF+5 new ones). Isolines image azimuthal gap. The new station sites are marked with *small circles*. Other abbreviations and explanations as in Fig. 11

**Fig. 13:**
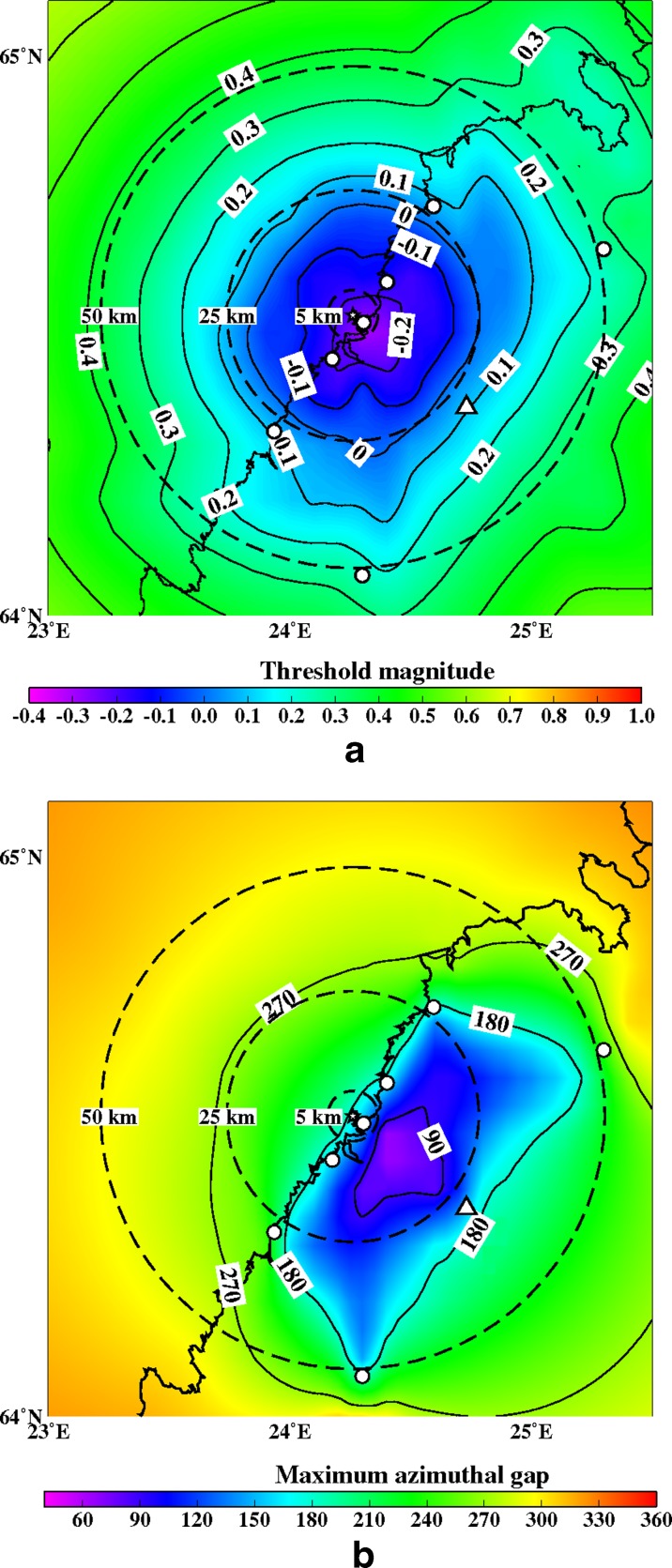
**a** The minimum detectable magnitude for the study region with an eight station network (OUF+7 new ones). Abbreviations and explanations as in Figs. 11 and 12a. **b** The maximum azimuthal gap for the study region with an eight station network (OUF+7 new ones). Abbreviations and explanations as in Figs. 11 and 12b

**Fig. 14:**
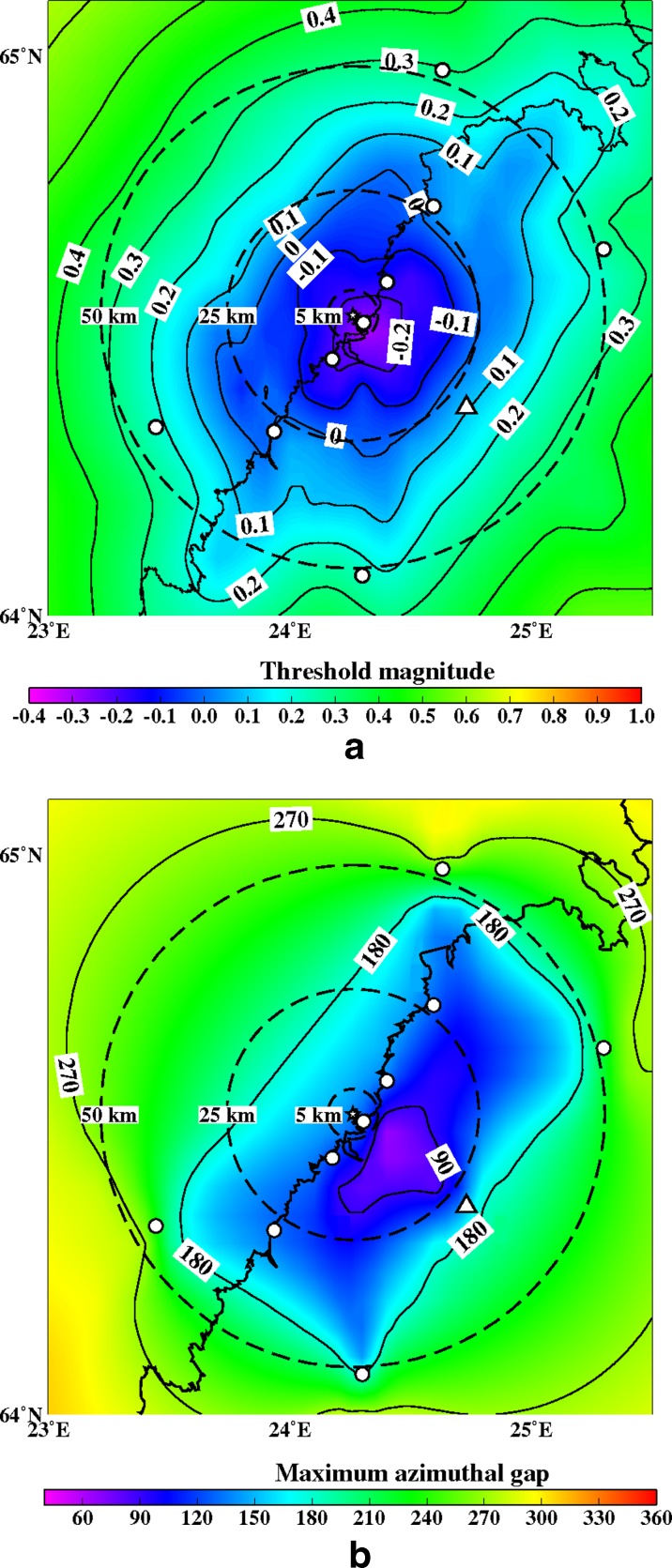
**a** The minimum detectable magnitude for the study region with a ten station network (OUF+9 new ones). Abbreviations and explanations as in Figs. 11 and 12a. **b** The maximum azimuthal gap for the study region with a ten station network (OUF+9 new ones). Abbreviations and explanations as in Figs. 11 and 12b

**Fig. 15:**
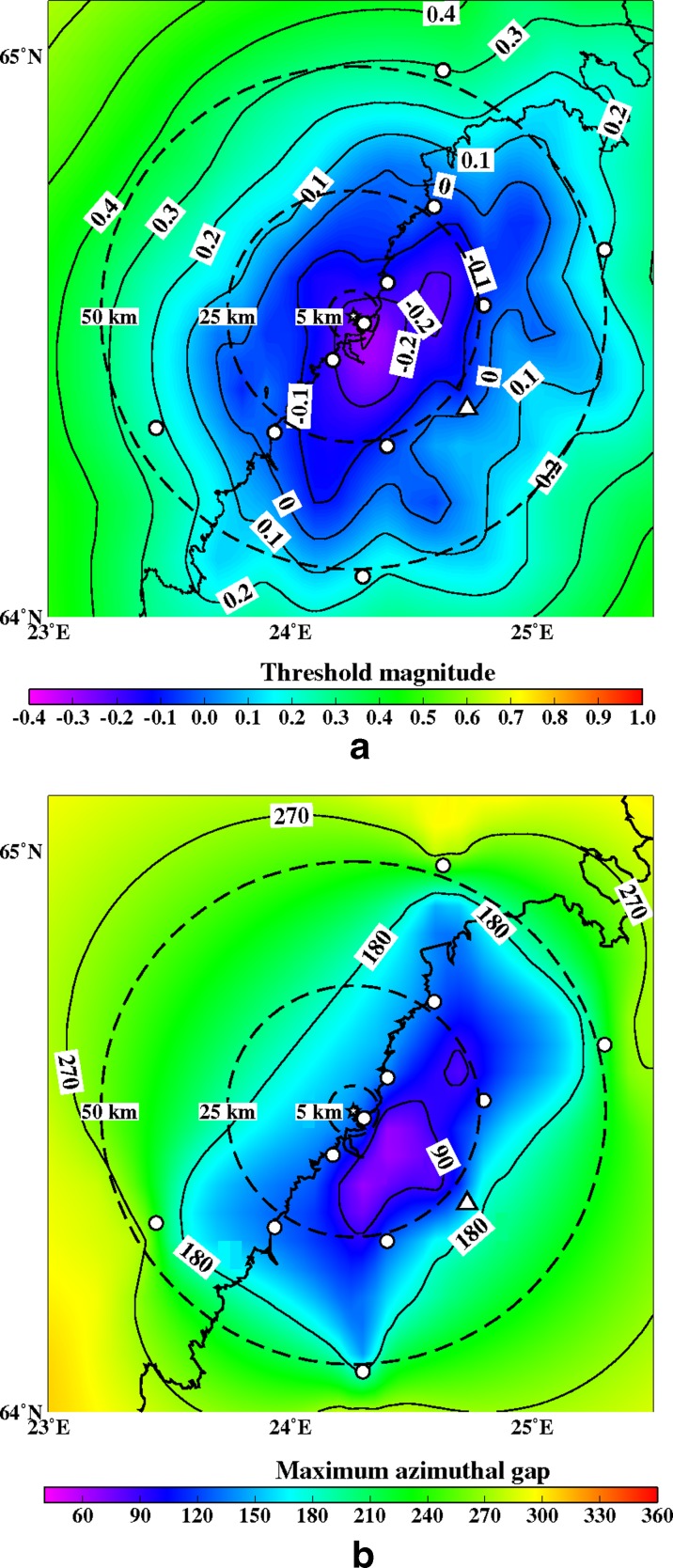
**a** The minimum detectable magnitude for the study region with a 12 station network (OUF+11 new ones). Abbreviations and explanations as in Figs. 11 and 12a. **b** The maximum azimuthal gap for the study region with a 12 station network (OUF+11 new ones). Abbreviations and explanations as in Figs. 11 and 12b

**Fig. 16:**
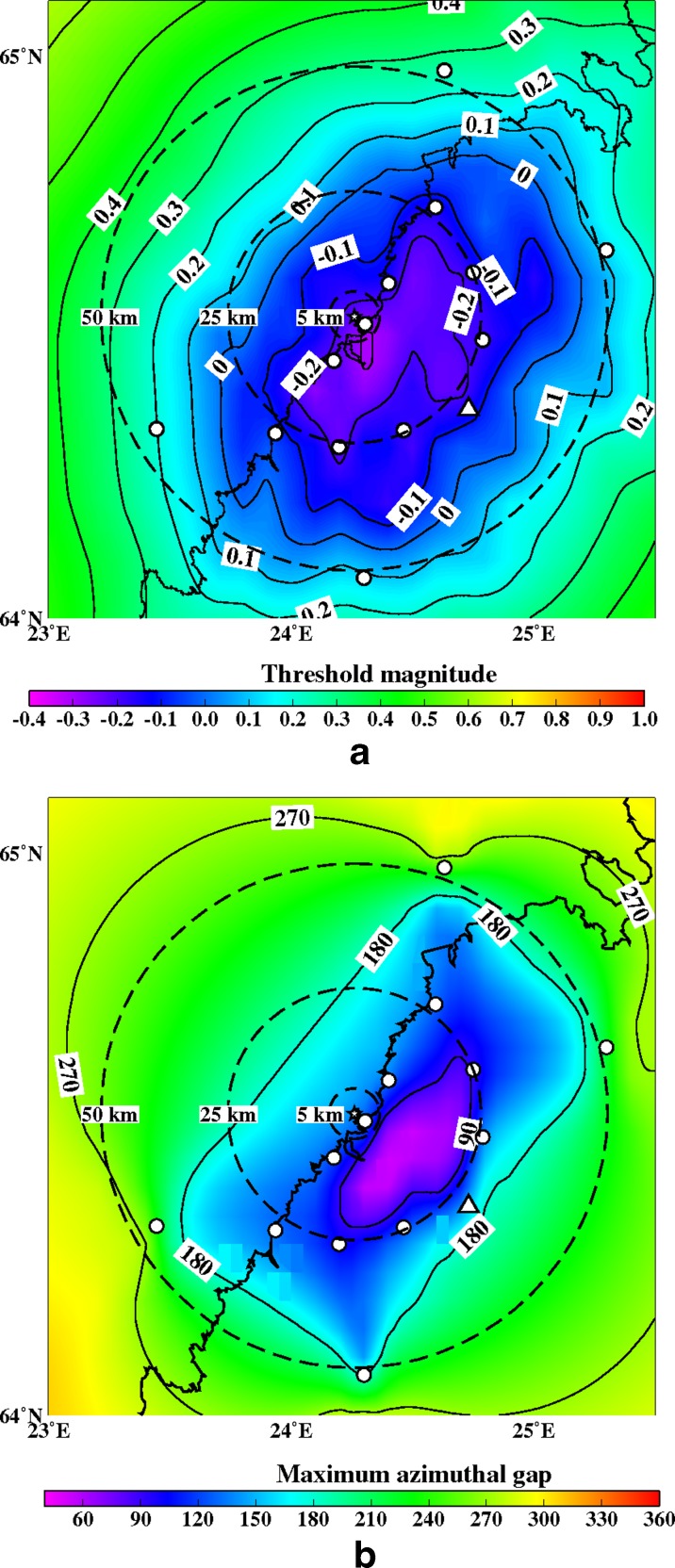
**a** The minimum detectable magnitude for the study region with a 14 station network (OUF+13 new ones). Abbreviations and explanations as in Figs. 11 and 12a. **b** The maximum azimuthal gap for the study region with a 14 station network (OUF+13 new ones). Abbreviations and explanations as in Figs. 11 and 12b

**Fig. 17:**
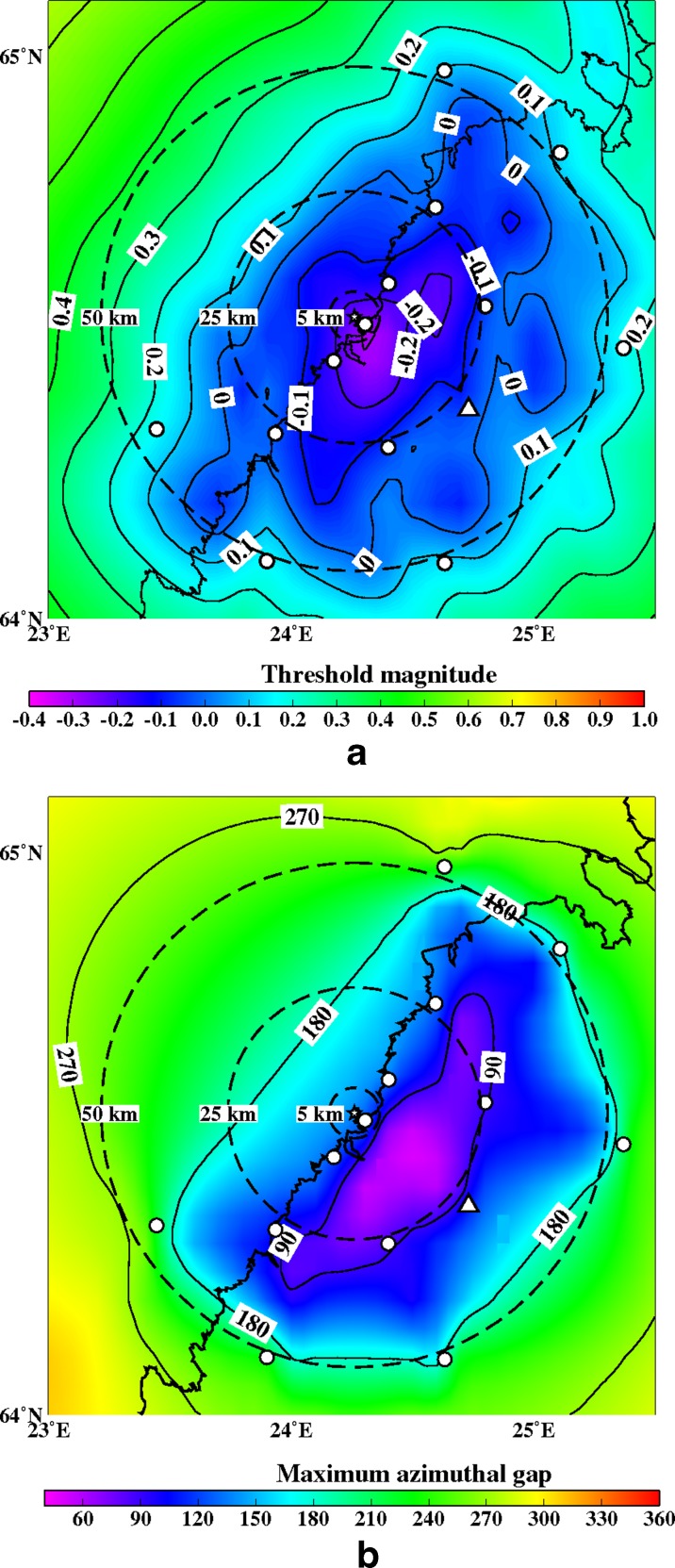
**a** The minimum detectable magnitude for the study region with a 14 station network (OUF+13 new ones), a different geometric configuration. Abbreviations and explanations as in Figs. 11 and 12a. **b** The maximum azimuthal gap for the study region with a 14 station network (OUF+13 new ones). Abbreviations and explanations as in Figs. 11 and 12b

The maps are calculated by forming a 0.1 × 0.1 degree grid over the area. Every grid point is a possible earthquake epicenter from which the distances to the stations are calculated. The distance used in the simulations is the distance to the third closest station of the simulated network at any point. This ensures that there will be phase readings from at least three stations, as required by ISUH automatic location process. This epicentral distance is then converted to the minimum detectable magnitude at the grid point by using Eq. (4). The simulations are aimed at achieving a good detection capability within 25 km radius of Pyhäjoki.

Another demand for Pyhäjoki network is a good azimuthal coverage within the 25 km radius area. The maximum azimuthal gap is directly related to network geometry and provides a quantitative measure on how consistently an event is surrounded by stations. It is one of the most useful criteria in the estimation of location accuracy (Bondár et al. 2004). Thus, maximum azimuthal gap is calculated at every point of the grid. A good azimuthal coverage could be gained by simply surrounding the area of interest with stations consistently. This demand cannot be fully met offshore and thus the azimuthal coverage will remain poorer offshore than onshore.

Each simulation of the minimum detectable magnitude involves also the closest surrounding stations available from the existing networks of FNSN and SNSN, although they are not displayed on the simulation maps. In the maximum azimuthal gap calculations, only those stations visible on the maps are used. This shows the realistic azimuthal coverage for events with minimum detectable magnitude, which are hardly seen by far-off stations. The results are presented in Figs. 11, 12, 13, 14, 15, 16, and 17b.

Figures 12a, 13, 14, 15, 16, and 17b show the simulation results for 7 different station configurations comprising 6–14 stations. The average threshold magnitude and azimuthal coverage of the networks are summarized in Table 2. The network consisting of six stations (Fig. 12a, b) has the capability to detect earthquakes down to magnitude 0.0 onshore within 25 km from the site. Offshore, the detection capability is slightly worse up to magnitude 0.3. The azimuthal gap is less than 180° onshore but 200°–270° offshore within 25 km from the site. The network with six stations has three stations on the coast, one station 25 km from the Pyhäjoki site, and two stations 50 km from the Pyhäjoki site. Figure 13a, b show the results of the eight stations network with two more stations near the coast. The location of Pyhäjoki at the shoreline sets limitations for the design of an equally spaced network over the whole study area. The denser network at the shoreline gives more uniform detection capability for both onshore and especially for offshore areas within 25 km distance of Pyhäjoki. The azimuthal gap in offshore area remains about the same as with the six stations network. In onshore area the azimuthal gap is smaller especially in the area 25–50 km from the Pyhäjoki site. The stations farther inland improve the azimuthal coverage. The next simulated network shown in Fig. 14a, b consists of 10 stations. The two additional stations are placed on the islands of Ulkokalla (south-west from Pyhäjoki) and Hailuoto (north from Pyhäjoki). In this case, the offshore detection capability has improved. Within 25 km offshore from the site, the detection threshold magnitude is 0.1 or smaller. Onshore, the detection thresholds remain about the same compared to the eight station network. The azimuthal gap is smaller than 180° for most of the area within 25 km radius. The largest azimuthal gap within 25 km from the site is only 193°. In the area 25–50 km from the Pyhäjoki site the azimuthal gap is clearly smaller than with the 8 station network. By increasing the number of stations to 12 (Fig. 15a, b) the detection capability improves onshore. There are no significant changes in azimuthal gap compared to Fig. 14b. The two added stations are located onshore about 25 km from the Pyhäjoki site. Thus, their effect is minimal on offshore area. Figures 16a, b and 17a, b show results from two different network configurations with 14 stations. The difference between networks in Fig. 16a, b compared to networks in Fig. 17a, b is that in the first network (Fig. 16a, b) the added two stations are 25 km from the Pyhäjoki site and in the latter network (Fig. 17a, b) they are 50 km from Pyhäjoki. The network in Fig. 16a, b improves the detection capability but the azimuthal gap does not improve compared to 12 station network. The network in Fig. 17a, b improves the azimuthal coverage significantly but the detection capability improves only slightly compared to 12 station network. In Fig. 16a, a detection threshold of magnitude −0.2 or better is obtained in over 50 % of the onshore area within 25 km from Pyhäjoki. This network shows the best detection capability of all tested networks. In Fig. 17b, the azimuthal gap is smaller than 180° within the offshore area less than 50 km from Pyhäjoki. This network shows the smallest azimuthal gaps of all simulated networks and thus the best location accuracy. Offshore, the situation cannot be improved without ocean bottom seismometers due to the lack of suitable islands for station installation. Adding new onshore stations has not improved the detection threshold or the azimuthal gap offshore compared to the network of 10 stations. Onshore, the average detection threshold has improved about 0.1 magnitudes in the area within 25 km from Pyhäjoki. Farther from Pyhäjoki, the improvement is more clear. In terms of azimuthal gap, the improvement of networks with more than 10 stations is observed mostly on onshore areas more than 25 km from Pyhäjoki. A uniform detection capability for earthquakes M_L_ ≥ 0.0 for onshore areas and offshore on the average within 21 km from the Pyhäjoki site can be obtained with a network of 10 stations.

**Table 2:** Comparison of the simulated networks and FNSN

	FNSN	Pyhä 6	Pyhä 8	Pyhä 10	Pyhä 12	Pyhä 14 v.1	Pyhä 14 v.2
Mt 5 km	0.90	0.03	−0.26	−0.26	−0.26	−0.26	−0.26
Mt 25 km	0.90	0.04	−0.08	−0.08	−0.10	−0.13	−0.10
Mt 50 km	0.88	0.21	0.15	0.12	0.08	0.04	0.05
A 90° 5 km	0 %	0 %	0 %	0 %	0 %	0 %	0 %
A 90° 25 km	0 %	6 %	17 %	17 %	20 %	26 %	26 %
A 90° 50 km	0 %	1 %	4 %	4 %	5 %	6 %	12 %
A 180° 5 km	0 %	50 %	50 %	100 %	100 %	100 %	100 %
A180° 25 km	0 %	46 %	46 %	89 %	89 %	89 %	89 %
A 180° 50 km	0 %	12 %	27 %	53 %	53 %	53 %	68 %

Average threshold magnitude (Mt) and azimuthal coverage (A) within a radius of 5, 25 and 50 km from Pyhäjoki are summarized. The simulated networks of 6–12 stations are named “Pyhä 6” etc. The two different network configurations of 14 stations in Fig. 16a, b and in Fig. 17a and b are marked with “Pyhä 14 v.1” and “Pyhä 14 v.2”, respectively

## Discussion

The simulation results show that a ten-station network gives a good detection capability within 25 km radius of Pyhäjoki. Another demand for Pyhäjoki network is good location accuracy. The azimuthal coverage of the stations has strong influence on the location accuracy. The simulated ten-station network gives a good azimuthal coverage within 25 km radius from Pyhäjoki. From Table 1, we see that, within a radius of 25 km from the plant site, we can expect to record two earthquakes (ML ≥ ∼ −0.1) per year and, from an area within a radius of 50 km, 5 events (ML ≥ ∼ −0.1 to ∼0.1) a year with a network of 10 stations. The occurrence rate of earthquakes with M_L_ ≥ 2.0 is one earthquake in 10 years within a radius of 50 km.

In the following, the location accuracy of the recommended seismic network of 10 stations will be estimated by comparing it with other networks in Fennoscandia and UK. Good azimuthal station coverage, sufficient number of seismic stations and short event-station distances are a prerequisite for accurate hypocenter determination (e.g., Bondár et al. 2004; Korja et al. 2010; Uski et al. 2011). The location accuracy is also affected by reading errors and the applied velocity model. Because the uncertainties in event location cannot be derived directly from the geometry of a network, examples from comparable set ups in geologically comparable areas are studied. In estimating future location accuracy, we use results from two national networks, FNSN (Korja et al. 2015) and SNSN (Bödvarsson et al. 2006, 2012), and from three local networks, KULN (Uski et al. 2011), Pärvie (Lindblom 2011), and Posiva (Saari and Malm 2010). The three local seismic networks have been operating on the Fennoscandian Shield during the last decade.

The relatively sparse FNSN (Fig. 4) provides an average epicenter location uncertainty of 3–5 km (Korja et al. 2010) and threshold magnitude of ∼0.9 (Fig. 11) within a radius of 25 km from Pyhäjoki. The FNSN automatic processing system uses data from 32 stations. The average station spacing of the FNSN is about 100 km. In SNSN standard processing, the median epicentral uncertainty is ca. 2 km and the threshold magnitude is 0.5. The SNSN consists of 66 seismic stations (Bödvarsson et al. 2012) and it has a station spacing of 66 km.

A dense local seismic network (Posiva) has been operating around the underground rock characterization facility in Olkiluoto, south-western Finland since 2002. The network of five stations is equipped with three component 1-Hz geophones suitable for investigating tectonic seismicity (Saari and Malm 2010). Within Olkiluoto and its surroundings, the threshold magnitude is approximately ML −1.0 and the horizontal location error less than 0.2 km (Saari, pers. comm., 2011).

Seismically active Kuusamo area has been monitored with a temporary network KULN since 2003. In its present composition, the network consists of one on-line broad-band station (KU6, see Fig. 4) and five off-line short-period stations within a radius of ca. Fifty kilometers around the permanent broad-band station MSF. The data of station MSF of the University of Oulu is included in the FNSN automatic processing system. Uski et al. (2011) estimate that when the azimuthal gap is less than 160° and the event-to-station distances are less than 250 km, the epicenter location accuracy is 0.5 and 1–2 km for local explosions and earthquakes, respectively. Furthermore, the uncertainty of unconstrained focal depths is estimated to be 4 km.

A dense temporary network has been operating around Pärvie end-glacial fault in northern Sweden during 2007–2010 (Lindblom 2011). In addition to eight temporary stations deployed around the fault, data from seven permanent stations have been used in the seismic analyses. Within the network of temporary stations, Lindblom (2011) has estimated the threshold magnitude to be −0.5 and the hypocenters to have average horizontal and depth uncertainties of 1–2 and 4 km, respectively. The location uncertainties have been further reduced by relocation with PStomo_eq, a three-dimensional local earthquake travel time tomography code by Tryggvason et al. (2002). On average, the uncertainties of PStomo_eq locations are 0.5 km in horizontal directions and 1.5 km in depth, for events with good azimuthal coverage. The Pärvie network and the simulated Pyhäjoki networks have similar station spacing and thus the uncertainties in the standard event location within the networks should be of the same order, i.e. 1–2 and 4 km in horizontal and vertical directions, respectively.

Location statistics for the different networks are summarized in Table 3. It is worth noting that the estimates of magnitude threshold in Table 3 agree rather well with the maximum detection distances derived from Eq. (4) in Fig. 10. The slightly lower value reported for the Pärvie network is acquired by the usage of a waveform cross-correlation technique (see Lindblom 2011).

**Table 3:** Comparison of the seismic networks used in the study

Network	Number of stations	Median station spacing (km)	Threshold magnitude	Location uncertainty (km) within the network
FNSN 2011	22	96	0.9	3–5
Kuusamo 2011	6	40	0.5	1–2
Pärvie 2007-10	15	23	–0.5	1–2
SNSN 2011	63	66	0.5	2
Posiva 2011	5	4	−1.0	0.2
Pyhäjoki 10	10	21	−0.1	1–2

Location uncertainties are from standard event processing

If the aim of the network is to have good detection capability (an event detection threshold of magnitude 0.0 or lower and azimuthal gap smaller than 180°) within 25 km distance from the site then a minimum of 10 seismic stations will be required. The comparison with other networks indicates the horizontal location accuracy to be about 1–2 km within the network. With this type of station setting it is possible to map and acquire information on capable faults within 25 km radius of the site. With the estimated threshold magnitudes, Mt = −0.1 and Mt = 0.1 within a radius of 25 and 50 km from Pyhäjoki, respectively, and the annual number of earthquakes detected by the network is estimated to be 2 (ML ≥ ∼ −0.1) within 25 km radius and 5 (ML ≥ ∼ −0.1 to ∼0.1) within 50 km radius (Table 1.). The simulations are based on minimum detection threshold magnitude. On favorable conditions, a magnitude 0.5 earthquake can be detected at 200 km distance in the study area. Some events will be detected at distance and magnitude ranges which do not have complete detection records. This will increase total number of detected earthquakes in the area. After some earthquakes are detected, correlation detectors can be used to find smaller but similar events (Gibbons et al. 2007). Correlation detectors can give detection thresholds down to one magnitude unit lower than traditional STA/LTA detectors (Schaff 2008).

Thus, the network is also suitable for acquiring information on faults within a distance of 25–50 km from the site which is important in intraplate area. The location accuracy is decreasing close to the 50 km ring and the focal mechanism of potential future events may be ambiguous. Some extra stations will be needed if the network was to function at maximum capability full time and even during occasional mechanical collapses of the stations. The proposed station on the island of Ulkokalla is easily reached for maintenance only between April and October. If the station encountered technical problems over a winter period, waveform data from the station would be inaccessible for months. Another station on the island of Ulkokalla or Maakalla (4 km from Ulkokalla) would ensure a continuous data flow from offshore areas.

The depth range of earthquakes within radius of 50 km from Pyhäjoki during the last 50 years has been from 3 to 28 km and most of the earthquakes are located in depth range from 7 to 10 km. Within the 10 station network (Fig. 14a) the largest distance to the nearest 3 stations is 20 km but smaller in most of the area within 25 km radius from Pyhäjoki. The typical depth of an earthquake is estimated to be from 7 to 10 km or more. The exact accuracy of the depth is difficult to estimate. Results from other networks indicate that the accuracy of depth could be about 3–4 km. This has been deduced by comparing the ten-station network setup with the KULN and Pärvie networks and results obtained from these networks (Uski et al. 2011 and Lindblom 2011). In the KULN, the accuracy of depth is about 4 km (Uski et al. 2011) and typical station spacing about 40 km. The automatic source locations can be fine tuned later by manual review of arrival times and by the application of three dimensional velocity models or relative event location methods, such as PStomo_eq or HypoDD (Waldhauser and Ellsworth 2000; Ma and Eaton 2011). Lindblom (2011) has estimated the accuracy of depth determination to be 4 km in the Pärvie network area. The Pärvie network has 15 stations but the area is larger and the station spacing varies more in the Pärvie network than in the Pyhäjoki 10 station network. Also noise conditions affect to hypocentral accuracy. The Pyhäjoki network is in an area with more human activity than the KULN or Pärvie networks. The noise levels in Pyhäjoki network are probably higher. The Pärvie network consists partly of temporary stations which have more poor noise conditions than permanent seismic stations (Lindblom 2011). The Pyhäjoki network will consist of permanent seismic stations which usually are more carefully installed than temporary stations and thus tend to have lower noise levels.

The theoretical simulation is based on data from FNSN and KULN, for which the station sites have been carefully selected. The permanent stations lie on bedrock in quiet areas with minimum amount of seismic background noise. The selected station configuration of 10 stations in Fig. 14a does not present final network, but a planned network that will give satisfactory results according to the network simulation. The final locations will be selected after noise study has been made in the area. According to preliminary noise studies, the noise levels are comparable to other locations in Finland (Valtonen et al. 2012; Korja et al. 2010). Since the area has abundance of bed rock outcrops, seismometers of most stations can be installed on bed rock. The study area has peat production plants and some small quarries producing seismic background noise. The area has also windmill parks, which produce seismic noise. Locations of these facilities will be taken into account when selecting the final station sites.

The pass band of instrument response of the seismometers at the stations should be wide enough in order to record signals in the whole expected frequency range. This is especially important for source mechanism studies that make use of spectral or time domain amplitude ratios and for the studies of ground motion acceleration. The highest frequency can be expressed in terms of the corner frequency (*f*_c_), i.e. the point at which the high-frequency part of the earthquake spectra starts to decay. At epicentral distance of 100 km, the theoretical S-wave corner frequency as a function of event magnitude can be approximated as follows (e.g., Eaton 1977; Lee and Stewart 1981):

5 $$ \operatorname{l}og\left({f}_c\right)=2.1-0.5{M}_L $$

Theoretical corner frequencies of S-waves for different small local magnitudes (M_L_) are shown in Table 4. When selecting a high-frequency recording system for micro-earthquake monitoring, sampling rate is an important factor. The seismograph should be programmed to record the events in enough detail to accurately reproduce the whole earthquake spectrum. In general, the sampling rate should be 2.5 times the highest expected frequency. Because the corner frequencies for P-waves are generally higher than those for S-waves (e.g., Molnar et al. 1973) a sampling rate of 500 Hz would ensure that both P- and S-wave frequency spectra is recorded down to magnitude ∼0. However, earthquakes with magnitude close to the detection threshold of the network are generally too weak for thorough spectral analysis. Earthquakes of M_L_ = 0.2 have S-wave corner frequency around 100 Hz. For these and larger events, a sampling rate of 250 Hz will be adequate. It will also be more cost-effective when using real-time data transfer. We cannot rule out possibility of recording larger events than previously observed in the study area since locations of intraplate earthquakes are difficult to predict. Their return period is very long, especially in Precambrian crust, and they usually lack accompanying surface ruptures (Gangopadhyay and Talwani 2003). Even moderate-size earthquakes (M = 5–6) may produce signals with energy in frequencies down to 0.1 Hz or lower (Havskov and Alguacil 2004). The stations should be capable of recording frequency range between 0.01 and 100 Hz. In any case, the capability of the whole network should be evaluated after an operation of full year and later at regular intervals. If needed, the sampling rate may be increased later. A thorough method for evaluating the performance of hypocenter location of a seismic network has been presented by D’Alessandro et al. (2011). A similar method should be applied after the network has produced enough data for evaluation.

**Table 4:** Relation between theoretical S-wave corner frequency and sampling rate

Sampling rate (Hz)	fc (Hz)	M_L_
1000	400	−1.0
500	200	−0.4
315	126	0.0
250	100	0.2
200	80	0.4

The corresponding magnitudes are also calculated

The network should also include strong motion instruments (IAEA 3.32, 2010) because they may be the only instruments recording the intense shaking of large events during which regular seismograph may be off-scale. Both the high-frequency and the strong motion recorders should be 3-C devices (IAEA 3.32, 2010). The network should also have a maintenance plan and a plan for replacing stations during mechanical problems. Additional stations could be used to fill the azimuthal gaps caused by station shut-downs.

## Conclusions

Based on the IAEA (2010) documentation and output of this study, it is recommend that the network to be installed around Pyhäjoki should be dense enough to fulfill the requirements of azimuthal coverage better than 180° and automatic event location capability down to M_L_ ∼ 0 within a distance of 25 km from the site.

One seismograph station, including 3-C high-frequency and strong motion seismographs (accelerographs), should be deployed in the site area. In addition, the network should comprise at least nine high-frequency 3-C stations within a radius of 50 km from the site.

With this setup the threshold magnitudes are estimated to be Mt = −0.1 and Mt = 0.1 within a radius of 25 and 50 km from Pyhäjoki, respectively. The annual number of earthquakes detected by the network is estimated to be 2 (ML ≥ ∼ −0.1) within 25 km radius and 5 (ML ≥ ∼ −0.1 to ∼0.1) within 50 km radius.

Within a 25-km distance from Pyhäjoki, the earthquake location accuracy is anticipated to be 1–2 and 4 km for horizontal coordinates and depth, respectively. It can be further improved by the application of local velocity models and relative location schemes.

A sampling rate of 250 Hz is recommended because it enables both cost efficient real-time data transfer and estimation of micro-earthquake spectrum down to M_L_ = 0.2.

It is recommended to link the data processing, analysis and reporting to the national analysis procedure, and thus the recordings of the stations should be publicly available on-line.

The theoretical network simulations do not take into account local factors, such as seismic noise sources, geology and infrastructure, which limit the number of sites available for good-quality seismic stations.
